# Combined Value of Red Blood Cell Distribution Width and Global Registry of Acute Coronary Events Risk Score for Predicting Cardiovascular Events in Patients with Acute Coronary Syndrome Undergoing Percutaneous Coronary Intervention

**DOI:** 10.1371/journal.pone.0140532

**Published:** 2015-10-15

**Authors:** Na Zhao, Lan Mi, Xiaojun Liu, Shuo Pan, Jiaojiao Xu, Dongyu Xia, Zhongwei Liu, Yong Zhang, Yu Xiang, Zuyi Yuan, Gongchang Guan, Junkui Wang

**Affiliations:** 1 Department of Cardiovascular Medicine, Shaanxi Province People’s Hospital, Xi’an, Shaanxi, China; 2 Department of Cardiovascular Medicine, First Affiliated Hospital of Medical College, Xi’an Jiaotong University, Xi’an, Shaanxi, China; 3 Department of Medical Affairs, Key Laboratory of Carcinogenesis and Translational Research (Ministry of Education), Peking University Cancer Hospital & Institute, Beijing, China; Medstar Washington Hospital Center, UNITED STATES

## Abstract

Global Registry of Acute Coronary Events (GRACE) risk score and red blood cell distribution width (RDW) content can both independently predict major adverse cardiac events (MACEs) in patients with acute coronary syndrome (ACS). We investigated the combined predictive value of RDW and GRACE risk score for cardiovascular events in patients with ACS undergoing percutaneous coronary intervention (PCI) for the first time. We enrolled 480 ACS patients. During a median follow-up time of 37.2 months, 70 (14.58%) patients experienced MACEs. Patients were divided into tertiles according to the baseline RDW content (11.30–12.90, 13.00–13.50, 13.60–16.40). GRACE score was positively correlated with RDW content. Multivariate Cox analysis showed that both GRACE score and RDW content were independent predictors of MACEs (hazard ratio 1.039; 95% confidence interval [CI] 1.024–1.055; p *<* 0.001; 1.699; 1.294–2.232; p *<* 0.001; respectively). Furthermore, Kaplan–Meier analysis demonstrated that the risk of MACEs increased with increasing RDW content (p *<* 0.001). For GRACE score alone, the area under the receiver operating characteristic (ROC) curve for MACEs was 0.749 (95% CI: 0.707–0.787). The area under the ROC curve for MACEs increased to 0.805 (0.766–0.839, p = 0.034) after adding RDW content. The incremental predictive value of combining RDW content and GRACE risk score was significantly improved, also shown by the net reclassification improvement (NRI = 0.352, p *<* 0.001) and integrated discrimination improvement (IDI = 0.023, p = 0.002). Combining the predictive value of RDW and GRACE risk score yielded a more accurate predictive value for long-term cardiovascular events in ACS patients who underwent PCI as compared to each measure alone.

## Introduction

Accurate risk stratification of patients with acute coronary syndrome (ACS) is important to efficiently target the use of evidence-based therapies and to identify high-risk patients who may benefit from advanced treatments. A multicenter registry recognized that the Global Registry of Acute Coronary Events (GRACE) risk score is a validated and established measure for stratifying patients with ACS according to risk and to guide treatment management decisions [1–3]. The clinical and laboratorial variables used by this risk scoring system include heart rate, systolic blood pressure, serum creatinine, and troponin. However, this system reflects only certain pathophysiological dimensions related to outcomes in ACS; biomarkers that addressed separate aspects of ACS pathophysiology could provide additional information. As reported in recent research, combining biochemical indices with the GRACE risk scoring system is better able to predict future cardiovascular events in patients with ACS as compared to the use of either measure alone [4–7].

Recently, considerably large clinical datasets have found that increased red blood cell distribution width (RDW) was a strong independent predictor of cardiovascular events in patients with heart diseases including ACS [8–12]. RDW represents the coefficient of variation in red blood cell volume distribution width. A variety of mechanisms, including inflammatory stress, neurohormonal pathways and adrenergic activation, nutritional deficiencies, and/or disordered iron homeostasis have been proposed to affect RDW [13–16]; however, these mechanisms were not considered in the GRACE risk scoring system. The combined value of RDW and GRACE score for predicting prognosis in ACS patients undergoing percutaneous coronary intervention (PCI) had never been assessed. Therefore, we studied the significance of adding the RDW to the GRACE score for use as a combined predictor.

In the present study, we investigated the individual value of RDW content and GRACE score for predicting major adverse cardiac events (MACEs) in patients with ACS undergoing PCI. We also studied the incremental prognostic value of combining RDW content with the GRACE score.

## Methods

### Study Cohort

We performed an observational study of consecutive patients with ACS who underwent PCI with stenting for the first time in the Shaanxi Province People’s Hospital and the First Affiliated Hospital of Xi’an Jiaotong University from December 2010 to January 2012. We included patients diagnosed with any of the ACS spectrum disorders, including unstable angina, non–ST-segment elevation myocardial infarction (NSTEMI), and ST-segment elevation MI (STEMI). Exclusion criteria were as follows: moderate to severe anemia (hemoglobin<90g/l) [17], history of PCI or coronary artery bypass graft (CABG), no stent implantation, bare metal stent (BMS) implantation, valvular heart disease, idiopathic dilated or hypertrophic cardiomyopathy, advanced liver disease, renal failure, cancer, stroke, peripheral arterial disease, pregnancy, use of anti-inflammatory drugs, acute or chronic infections or autoimmune disease, and malignant blood disease or thyroid disease.

The study complied with the Declaration of Helsinki and was approved by the ethics committee of the 2 hospitals mentioned above. Written consent was obtained from all patients.

### Demographic and clinical data

Demographic data and cardiovascular risk factors were obtained from the medical records. Current smokers were defined as having smoked >100 cigarettes during their lifetime and as those that had smoked within the previous 30 days.

### Blood samples and echocardiography

Peripheral blood was sampled from patients in a fasting state on the morning following the admission day. Venous plasma concentrations of glucose, lipids, lipoproteins, serum creatinine, HbA1c, N-terminal pro-B-type natriuretic peptide (NT-proBNP), white blood cells, platelets, and RDW content were determined in the clinical laboratory department using standard biochemical techniques. Echocardiographic data (left ventricular ejection fraction [LVEF]) was measured using Doppler echocardiography performed within 3 days of admission.

### Calculation of GRACE risk score

The GRACE risk scoring system has been previously described [2]. The score is derived from several variables (age, heart rate, systolic blood pressure, creatinine level, congestive heart failure, in-hospital percutaneous coronary intervention, in-hospital coronary aortic bypass grafting, history of myocardial infarction, ST-segment depression, and elevated cardiac enzyme/marker levels) and calculated for each patient. The GRACE risk score was originally designed to predict mortality 6 months after discharge, and it has been shown to have good predictive value for mortality up to 4 years after an ischemic event [1–2,18].

### Outcomes and follow-up

MACEs were defined as all-cause death or nonfatal myocardial infarction. The non-fatal myocardial infarction was defined as an at least 2 times of elevation of the creatine kinase-myocardial band (CK-MB) compared to the normal upper limit or a Q-wave myocardial infarction [19]. All patients were followed up by the 2 hospitals with face-to-face interviews or telephone contact. The follow-up end point was defined as the date of first MACE occurrence, obtained via reviewing hospital records. Some patients were followed up until December 2014.

### Statistical analysis

Data were collected using IBM SPSS statistical software version 20.0 for Windows (SPSS Inc., Chicago, IL). Continuous variables were expressed as the mean and standard deviation. Categorical variables were expressed as frequencies and percentages. The Kolmogorov–Smirnov test was used to assess the normal distribution of quantitative variables. The independent samples *t*-test was performed to compare parametric values between the MACE group and the non-MACE group, whereas categorical variables were compared using the Chi-square test. One-way ANOVA was performed to compare the parametric values among multiple groups. Univariate and multivariate survival analyses were performed using Cox regression. To further assess the prognostic value of RDW, Kaplan–Meier survival curves were used in the 3 groups divided according to the RDW level. The predictive values of RDW and a combination of RDW and GRACE risk score were estimated by comparing the areas under the receiver operating characteristic (ROC) curve. DeLong's test was used to compare the AUC from each of models[20], which were analyzed by use of MedCalc Version 11.4.2.0. Additionally, the increased discriminative value after the addition of RDW to the GRACE was also estimated using 2 measures (the net reclassification improvement [NRI], and integrated discrimination improvement [IDI]). The IDI was equal to the increase in discrimination slope defined as the mean difference in predicted risks between those with and without events. The continuous NRI was a non-parametric analogue of the IDI and equals twice the difference in probabilities of upward reclassification for events minus for non-events[21–22]. Statistical analysis was performed using IBM SPSS Statistics 20.0 for windows (SPSS Inc., Chicago, IL), and R (version i386 3.2.1 for Windows). All probability values were 2-tailed. A p value of <0.05 was considered statistically significant.

## Results

### Baseline characteristics of patients

The 480 patients included in this study were divided into 3 groups (tertiles) according to the baseline RDW content (tertile 1: 11.30–12.90; tertile 2: 13.00–13.50; tertile 3: 13.60–16.40). Patient demographics and other clinical characteristics are shown in Table 1. Intergroup comparisons revealed that GRACE scores increased along with higher RDW levels (p < 0.001). The systolic blood pressure (SBP) and HbA1c levels were lower in tertile 3 than in tertiles 1 and 2 (p = 0.02 and p = 0.009, respectively). The N-terminal pro-B-type natriuretic peptide (NT-proBNP) and creatinine levels of patients in tertiles 1 and 2 were significantly lower (p = 0.003 and p = 0.001, respectively) than they were in patients in tertile 3. Hemoglobin (HGB) levels and mean corpuscular hemoglobin concentration (MCHC) were lower in the presence of a higher RDW (p = 0.029 and p = 0.009, respectively). Low-density lipoprotein cholesterol (LDL) in tertile 1 was higher than that in tertiles 2 and 3 (p = 0.043). No difference was observed among the 3 groups in other baseline characteristics.

**Table 1:** Baseline characteristics of 480 patients with acute coronary syndrome (ACS) undergoing percutaneous coronary intervention (PCI) according to the RDW content tertiles.

Variable	Tertile 1	Tertile 2	Tertile 3	P value
	11.30–12.90	13.00–13.50	13.60–16.40	
	n = 159	n = 160	n = 161	
GRACE score	86.18 ± 25.09	96.15± 27.76	99.72 ± 26.00	**< 0.001**
Age (years)	59.92 ± 9.35	61.03 ± 11.29	62.35 ± 10.56	0.113
Male	111 (69.8)	121 (75.2)	120 (74.5)	0.459
BMI (kg/m^2^)	24.10 ± 2.91	24.14 ± 3.54	24.16 ± 3.13	0.985
Smoking	88 (55.3)	98 (60.9)	101 (62.7)	0.363
Hypertension	94(59.1)	92(57.5)	98(60.9)	0.828
Diabetes	63(39.6)	53(33.1)	58(36.0)	0.481
Hyperlipidemia	43(27.0)	29(18.1)	30(18.6)	0.092
Prior MI	14(8.8)	14(8.7)	9(5.6)	0.466
ACS				0.741
Unstable Angina	94(59.2)	92(57.1)	93(57.8)	
NSTEMI	15(9.4)	23(14.3)	20(12.4)	
STEMI	50(31.4)	45(28.1)	48(29.8)	
Heart rate (beats/min)	74.59 ± 13.66	72.79 ± 12.54	75.71 ± 16.66	0.188
SBP (mmHg)	131.10 ± 18.47	128.90 ± 18.45	125.21 ± 20.12	**0.020**
DBP (mmHg)	79.97 ± 10.91	79.86 ± 12.19	77.83 ± 13.19	0.209
FBG (mmol/L)	7.35 ± 3.30	7.02 ± 3.28	7.26 ± 3.28	0.646
HbA1c (%)	6.47 ± 1.56	6.13 ± 1.11	6.07 ± 0.97	**0.009**
Cystatin C (mg/L)	0.93 ± 0.40	1.04 ± 0.76	1.05 ± 0.41	0.091
Creatinine (umol/L)	71.11 ± 18.68	78.16 ± 22.71	82.71 ± 30.31	**0.001**
TC (mmol/L)	4.01 ± 0.97	3.90 ± 1.51	3.93 ± 1.82	0.799
HDL (mmol/L)	1.00 ± 0.27	1.01 ± 0.26	0.96 ± 0.26	0.244
LDL (mmol/L)	2.38 ± 0.77	2.19 ± 0.71	2.20 ± 0.82	0.043
In Lpa	5.08 ± 0.90	5.12 ± 0.85	5.14 ± 0.78	0.803
LVEF (%)				0.689
≥ 55	108	106	104	
45–54	27	27	29	
31–44	22	24	26	
≤ 30	2	3	2	
lnBNP	5.34 ± 1.61	5.62 ± 1.56	5.96 ± 1.70	**0.003**
WBC count (10^9^/L)	7.70 ± 2.96	7.56 ± 2.87	7.73 ± 2.94	0.846
PLT count (10^9^/L)	191.59 ± 54.92	185.82 ± 64.33	186.14 ± 77.41	0.682
RBC count (10^12^/L)	4.51 ± 0.54	4.46 ± 0.53	4.40 ± 0.64	0.260
HGB (g/L)	140.60 ± 14.71	138.34 ± 15.97	135.93 ± 17.74	**0.029**
MCV (fL)	92.20 ± 4.22	92.78 ± 4.80	92.93 ± 6.43	0.418
MCH (pg)	31.23 ± 1.57	31.24 ± 1.74	31.16 ± 3.05	0.944
MCHC (g/L)	339.56 ± 11.97	336.87 ± 10.44	334.91 ± 19.82	**0.009**
HCT (%)	40.97 ± 4.83	41.30 ± 4.38	40.95 ± 5.40	0.778
Aspirin	156 (98.1)	158 (98.8)	161 (100)	0.239
Clopidogrel	151 (95.0)	157 (98.1)	152 (94.4)	0.200
Statins	113 (71.1)	115 (71.9)	109 (67.7)	0.686
ACEI/ARB	114 (71.7)	120 (74.5)	113 (70.2)	0.677
β-blockers	111 (69.8)	120 (74.5)	112 (69.6)	0.540

Data are presented as mean ± SD or n (%). ACEI, angiotensin-converting enzyme inhibitor; ACS, acute coronary syndrome; ARB, angiotensin-receptor blocker; BMI, body mass index; SBP, systolic blood pressure; DBP, diastolic blood pressure; FBG, fasting blood glucose; HbA1c, hemoglobin A1c; HDL-C, high-density lipoprotein cholesterol; HGB, hemoglobin; LDL-C, low-density lipoprotein cholesterol; LPA, lipoprotein(a); LVEF, left ventricular ejection fraction; MCV, mean corpuscular volume; MCHC, mean corpuscular hemoglobin; MCHC, mean corpuscular hemoglobin concentration; NSTEMI, non-ST elevation myocardial infarction; NT-proBNP, N-terminal pro–B-type natriuretic peptide; PLT, platelets; RBC, red blood cell count; STEMI, ST elevation myocardial infarction; TC, total cholesterol; TG, triglycerides; WBC, white blood cell count.

### Comparison of clinical characteristics between patients with and without MACEs

During a median follow-up time of a 37.2 months (interquartile range, 37.0–42.2 months), 15 of the 495 subjects (3.0%) were lost to follow-up. Data from the 480 remaining patients were analyzed. Of these, 70 (14.58%) had a MACE, including 51 all-cause deaths and 19 acute myocardial infarctions. The baseline characteristics of patients with or without MACEs are outlined in Table 2. Compared with the patients without MACEs, those who experienced MACEs had lower MCHC levels; and had higher fasting blood glucose (FBG), HbA1c, and NT-ProBNP levels. In addition, LVEF was significantly related to MACE occurrence (p = 0.020). Moreover, both GRACE risk scores and RDW levels were higher in patients who experienced MACEs (p *<* 0.001 for both comparisons).

**Table 2:** Characteristics of acute coronary syndrome patients undergoing percutaneous intervention (PCI) with or without a major adverse cardiac event (MACE).

Variable	With MACE	Without MACE	p value
	n = 70	n = 410	
GRACE score	113.63 ± 26.87	90.70 ± 26.17	**< 0.001**
Age (years)	63.29 ± 11.45	60.73 ± 10.25	0.059
Male	52 (74.3)	300 (73.2)	0.885
BMI (kg/m^2^)	24.29 ± 3.29	24.11 ± 3.19	0.667
Smoking	45 (64.3)	242 (59.0)	0.432
Hypertension	45(63.4)	239(58.3)	0.346
Diabetes	26(36.6)	148(36.1)	0.886
Hyperlipidemia	8(11.3)	94(22.9)	**0.030**
Prior MI	9(12.9)	28(6.8)	0.081
ACS			0.670
Unstable Angina	44(62.9)	235(57.3)	
NSTEMI	8(11.4)	50(12.2)	
STEMI	18(25.7)	125(30.5)	
Heart rate (beats/min)	76.47 ± 17.26	74.01 ± 13.87	0.187
SBP (mmHg)	124.37 ± 19.16	129.08 ± 19.08	0.057
DBP (mmHg)	77.87 ± 13.36	79.45 ± 11.93	0.316
FBG (mmol/L)	8.30 ± 4.32	7.02 ± 3.04	**0.003**
HbA1c (%)	6.39 ± 1.03	6.19 ± 1.28	**0.037**
Cystatin C (mg/L)	1.08 ± 0.33	0.99 ± 0.58	0.194
Creatinine (umol/L)	82.49 ± 24.70	76.47 ± 24.76	0.061
TC (mmol/L)	3.74 ± 0.99	3.98 ± 1.54	0.196
HDL (mmol/L)	1.00 ± 0.28	0.99 ± 0.26	0.654
LDL (mmol/L)	2.09 ± 0.66	2.28 ± 0.78	0.057
In LPA	5.19 ± 0.96	5.09 ± 0.82	0.392
LVEF (%)			**0.020**
≥ 55	31	303	
45–54	26	57	
31–44	11	45	
≤ 30	2	5	
InBNP	6.35 ± 1.82	5.52 ± 1.58	**< 0.001**
WBC count (10^9^/L)	7.94 ± 3.03	7.62 ± 2.90	0.399
PLT count (10^9^/L)	184.67 ± 80.91	188.38 ± 63.40	0.665
RBC count (10^12^/L)	4.45 ± 0.68	4.46 ± 0.55	0.889
HGB (g/L)	136.50 ± 17.30	138.98 ± 16.10	0.240
MCV (fL)	93.24 ± 7.09	92.53 ± 4.97	0.296
MCH (pg)	30.95 ± 2.35	31.26 ± 2.20	0.289
MCHC (g/L)	331.75 ± 9.23	337.86 ± 15.36	**0.001**
HCT (%)	41.40 ± 5.48	41.02 ± 4.78	0.859
RDW content (per %)	13.86 ± 0.92	13.24 ± 0.81	**0.001**
Aspirin	70 (100)	405 (98.8)	0.353
Clopidogrel	66 (94.3)	394 (96.1)	0.483
Statins	46 (65.7)	291 (71.0)	0.974
ACEI/ARB	47 (67.1)	300 (73.2)	0.298
β-blockers	53 (75.7)	289 (70.5)	0.372

Data are expressed as mean ± SD or n (%). GRACE, Global Registry of Acute Coronary Events, RDW, red blood cell distribution width.

### Correlation of GRACE risk score with RDW

The correlations between GRACE risk score and RDW were analyzed using Spearman’s rank correlation. The results showed that GRACE risk score was significantly and positively correlated with RDW (R = 0.259, p *<* 0.001).

### RDW as an independent predictor of MACE

**Fig 1:**
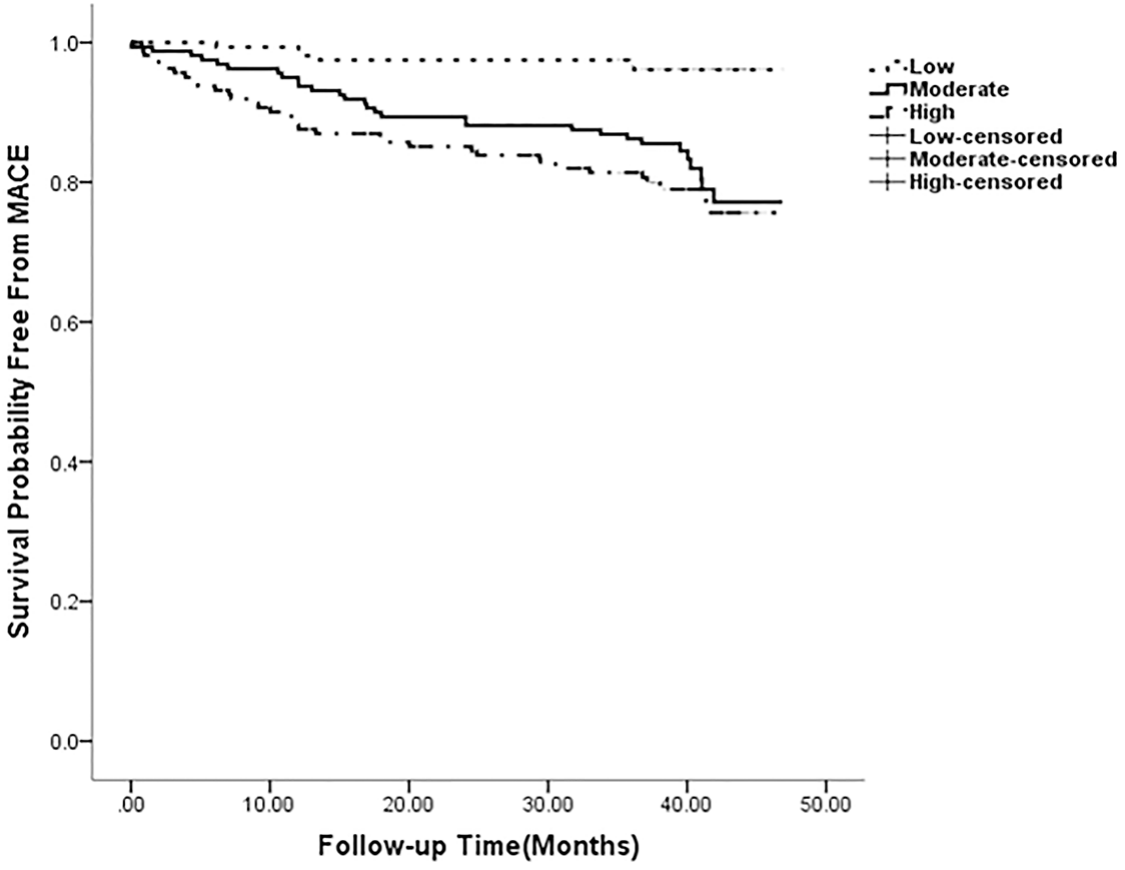
Kaplan–Meier analysis of major adverse cardiac events based on red blood cell distribution width. The 480 patients of 70 had a MACE were divided by tertiles based on the RDW (tertile 1: 11.30–12.90, tertile 2: 13.00–13.50, and tertile 3: 13.60–16.40). The risk of MACE increased along with increasing RDW (p < 0.001).

After performing univariate Cox analysis, significant predictors MACE were hyperlipidemia, higher levels of FBG, HbA1c, NT-ProBNP, GRACE risk score, and RDW, and lower levels of MCHC, and LVEF (Table 3). Table 4 presents the results of multivariate Cox analysis, which demonstrated that RDW was a significant and independent predictor of MACEs (HR: 1.699; 95% CI: 1.294–2.232; p *<* 0.001). In addition, GRACE risk score (HR: 1.039; 95% CI: 1.024–1.055; p *<* 0.001) and FBG (HR: 1.076; 95% CI: 1.018–1.138; p = 0.010) were identified as significant predictors of MACEs. Kaplan–Meier analysis indicated the estimated cumulative incidence of MACEs, as seen in Fig 1. The possibility of experiencing a MACE increased along with an increase in RDW. A log-rank test of the curves for the 3 patient groups identified significant intergroup differences (p *<* 0.001).

**Table 3:** Univariate Cox analysis for major adverse cardiovascular events.

Variable	HR	95% CI	P value
GRACE Score (per 1 point)	1.033	1.023–1.042	**<0.001**
Age (per year)	1.022	0.999–1.046	0.065
Male	1.075	0.629–1.837	0.792
BMI (per kg/m^2^)	1.016	0.944–1.093	0.677
Smoking	1.224	0.751–1.996	0.417
Hypertension	0.797	0.489–1.299	0.363
Diabetes	0.949	0.584–1.541	0.832
Hyperlipidemia	2.153	1.031–4.497	**0.041**
Prior MI	0.532	0.264–1.070	0.077
Heart rate (per bpm)	1.010	0.995–1.024	0.197
SBP (per mmHg)	0.987	0.974–1.000	0.050
DBP (per mmHg)	0.990	0.971–1.010	0.340
FBG (per mmol/L)	1.090	1.031–1.152	**0.002**
HbA1c (per %)	1.106	0.943–1.296	**0.021**
Cystatin C (per mg/L)	1.186	0.932–1.509	0.166
Creatinine (per umol/L)	1.007	1.000–1.014	0.042
TC (per mmol/L)	0.842	0.661–1.073	0.166
HDL (per mmol/L)	1.209	0.504–2.898	0.671
LDL (per mmol/L)	0.726	0.522–1.010	0.057
In Lpa (per ln unit)	1.143	0.861–1.516	0.354
LVEF (per %)	0.974	0.957–0.992	**0.005**
InBNP (per ln unit)	1.351	1.170–1.560	**<0.001**
WBC (per 10^9^/L)	1.026	0.953–1.103	0.500
PLT (per 10^9^/L)	0.999	0.995–1.003	0.646
RBC (per 10^12/^L)	0.978	0.645–1.481	0.915
HGB (per g/L)	0.991	0.977–1.005	0.214
MCV(per fL)	1.022	0.977–1.069	0.343
MCH (per pg)	0.922	0.813–1.045	0.202
MCHC (per g/L)	0.960	0.941–0.980	**<0.001**
HCT (per %)	1.015	0.965–1.067	0.560
RDW (per %)	2.092	1.654–2.646	**<0.001**
Aspirin	0.049	0.000–3.154	0.542
Clopidogrel	1.473	0.537–4.043	0.452
Statins	1.327	0.808–2.181	0.264
β-blockers	0.796	0.461–1.375	0.414
ACEI/ARB	1.309	0.795–2.157	0.290

HR, hazard ratio; CI, confidence interval.

**Table 4:** Multivariate Cox analysis for major adverse cardiovascular events.

Variable	HR	95% CI	P value
GRACE score (per point)	1.039	1.024–1.055	**<0.001**
FBG (per mmol/L)	1.076	1.018–1.138	0.010
RDW (per %)	1.699	1.294–2.232	**<0.001**

### Effect of combining RDW and GRACE score in the prediction of MACE occurrence

**Fig 2:**
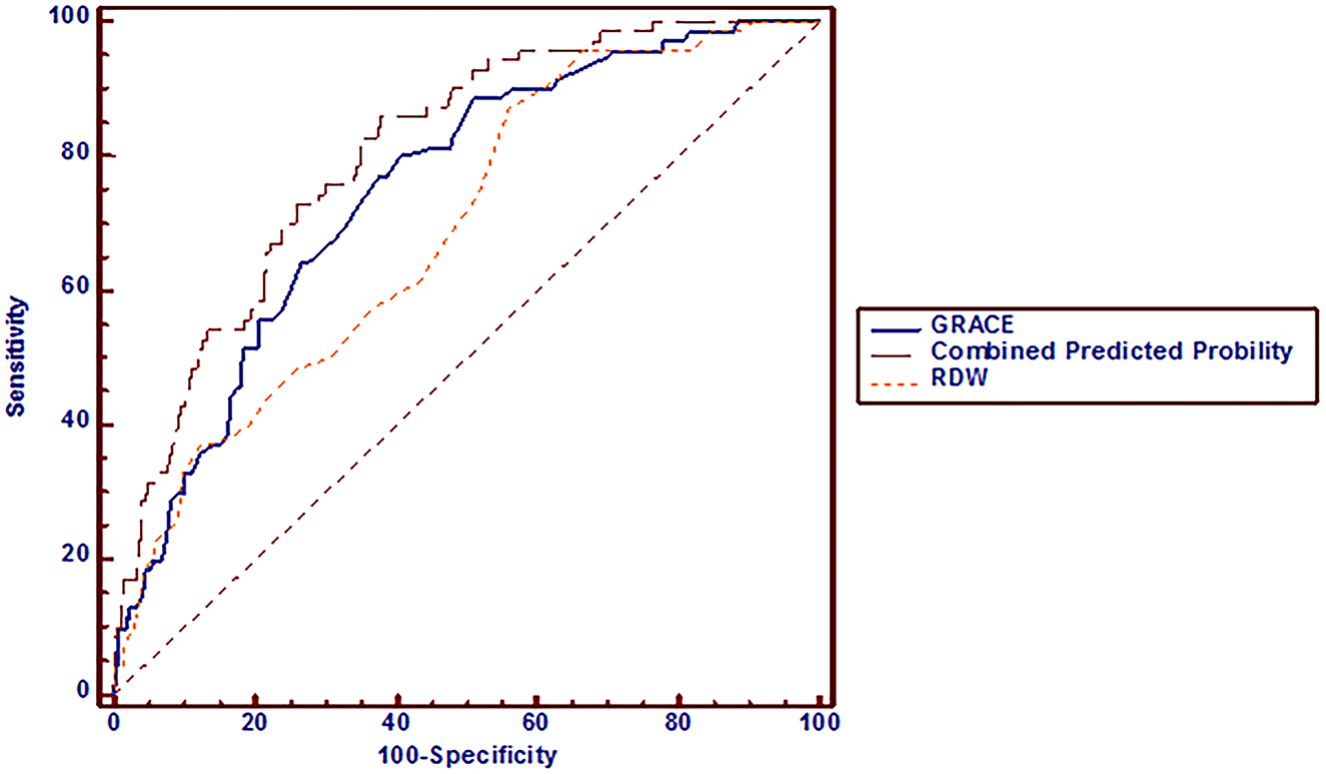
Receiver operating characteristic (ROC) curve analysis for predicting cardiovascular events. For GRACE score alone, the area under the curve (AUC) was 0.749 (95% CI: 0.707–0.787). When RDW was added to GRACE score, the AUC was 0.805 (95% CI: 0.766–0.839, p = 0.034).

Because both RDW and GRACE score were independent risk factors for MACEs, we assessed their combined value for predicting the long-term risk of MACE. For GRACE score alone, the area under the curve (AUC) was 0.749 (95% CI: 0.707–0.787). When RDW was added to GRACE score, the AUC was 0.805 (95% CI: 0.766–0.839, p = 0.034) (Fig 2). In addition, reclassification of patients with or without MACEs at the time of follow-up is presented in Table 5. The addition of RDW to GRACE score significantly improved the reclassification (0.352, p < 0.001) and the integrated discrimination (0.023, p = 0.002) of subjects compared to GRACE score system (Table 5). These results revealed that the predictive value of adding RDW to the GRACE score was superior to the predictive value of the GRACE score alone in predicting MACEs.

**Table 5:** Discrimination of model in predicting major adverse cardiovascular events (MACE).

Events, n (%)	70 (14.6)	
Nonevents, n (%)	410 (85.4)	
Continuous NRI		**p< 0.001**
cNRI events	0.211	
cNRI nonevents	0.141	
cNRI	0.352	
IDI statistics		**P = 0.002**
IDI events	0.010	
IDI nonevents	0.013	
IDI	0.023	

NRI, Net Reclassification Improvement; IDI, Integrated Discrimination Improvement

## Discussion

The prognostic value of GRACE risk score for making treatment decisions has been clearly demonstrated in patients with ACS. Current guidelines recommend using the GRACE score for risk stratification in these patients [23,24]. Our research showed that GRACE risk score can independently predict MACEs in ACS patients who underwent PCI. However, in accordance with the results of previous research, the present study found that the ability of this scoring system to discriminate among target groups still needs to be improved [3,25,26]. The scoring system might be limited partly because certain disease parameters, such as inflammation, oxidative stress, and nutritional deficiencies, are not fully captured by the system’s variables. As mentioned earlier, RDW has been proposed as a potential additional variable to this system. Indeed, both our study and a previous study demonstrated a significant correlation between RDW and the GRACE risk score [27], indicating that adding RDW to the GRACE score could enhance the predictive value of each measure in patients with ACS who underwent PCI.

Emerging evidence supports that the RDW in ACS patients (with or without a history of PCI) as an independent risk factor for future cardiovascular events [28,29]. The present results demonstrated that several cardiovascular risk factors and biochemical risk markers are positively correlated with RDW. In addition, oxidative stress, and/or nutritional deficiencies, shift work [30] was also found to be related to RDW. Thus, these underlying associations of RDW with the markers of cardiovascular disease burden could explain the prognostic value of RDW. Our study found that increasing levels of RDW were associated with an increased risk of MACE and verified that RDW could independently predict long-term MACEs in ACS patients who underwent PCI. Moreover, these results confirmed that RDW added discriminatory predictive value to the GRACE score. This added value was shown by the significant increase in AUC from 0.749 to 0.805 for the combined endpoint of death or nonfatal myocardial infarction in ACS patients who underwent PCI. In addition, discrimination of GRACE score adjustment by RDW variable was also powerfully certified by new statistical metrics (continuous NRI and IDI). We have found a net 14% of the patients without events were reclassified into lower risk and that a net 21% of patients with events were reclassified into higher risk. The continuous NRI thus reached an impressive 0.352, which suggested that the RDW content led to a significant net reclassification of patients^,^ risk in the appropriate directions. We also have found an IDI for RDW showed further average separation of events from non-events by the RDW. These results showed that the predictive value was improved by adding RDW to the model with GRACE risk score.

Currently, RDW is a widely included parameter in complete blood count analyses in most clinical laboratories. Thus, compared with other biomarkers of cardiovascular risk, RDW testing incurs no additional costs. Therefore, combining RDW with GRACE risk scoring could help evaluate long-term cardiovascular disease risk in patients with high-risk ACS who underwent PCI. Consequently, these predictive factors could improve patient outcomes and help in making treatment management decisions in clinical practice.

The present study showed that, in addition to the GRACE score, systolic blood pressure, NT-proBNP, creatinine, and LDL levels were also associated with RDW. These associations indicate that the underlying effects involving RDW on biochemical indices should be explored in subsequent research. Because HGB and MCHC are common factors affected by RDW, we found a negative correlation between RDW and HGB and MCHC levels, as described in previous reports [9,31].

Our study also found that FBG level was an independent predictor of MACE, regardless of diabetes status. This finding was consistent with those of previous publications [32,33], most of which revealed that FBG levels can predict in-hospital and short-term prognoses in ACS patients [34,35]. The exact relationship between FBG and cumulative MACE occurrence rate will need to be assessed in future research.

The current study has several limitations. The patient population only included Chinese individuals, and the number of patients was relatively small. Furthermore, the RDW values were not tested at the same laboratory. Additionally, the population was limited by ACS patients underwent PCI for the first time, this does not reflect the distribution in all ACS patients. Additional observations in larger-scale population will be needed to further elucidate the prognostic value of combining RDW with the GRACE risk scoring system.

## Conclusion

The present study showed that both the GRACE scoring system and RDW testing have an individual predictive value for cardiovascular events in ACS patients who underwent initial PCI. Moreover, these measures are independently and positively related to each other. Combining the 2 approaches resulted in higher predictive value for long-term cardiovascular events in ACS patients who underwent PCI.
